# Group-based rewiring rules of binary opinion competition dynamics

**DOI:** 10.1038/s41598-018-32678-7

**Published:** 2018-09-26

**Authors:** Cheng Jin, Chunji Yin, Xiaogang Jin, Yong Min, Yixiao Li, Nuole Chen, Jiaxuan Huang

**Affiliations:** 1 Institute of Artificial Intelligence, College of Computer Science & Technology, Zhejiang University, 310027 Hangzhou, China; 2 Tencent Technology (Shenzhen) Co., Ltd., 518057 Shenzhen, China; 3 State Key Lab of CAD&CG, Zhejiang University, 310058 Hangzhou, China; 4 College of Computer Science, Zhejiang University of Technology, 310023 Hangzhou, China; 5 School of Information, Zhejiang University of Finance and Economics, 310018 Hangzhou, China; 6 Department of Political Science, University of Illinois at Urbana-Champaign, 61820 Urbana, United States

## Abstract

The dynamics of competing opinions on networks has attracted multi-disciplinary research. Most modelling approaches assume uniform or heterogeneous behaviour among all individuals, while the role of distinctive group behaviour is rarely addressed. Here, we consider competition occurring between two opinion groups with bound rewiring rules, i.e., opinion-preferred rewiring, degree-preferred rewiring and random rewiring. When two opinions share a balanced initial proportion, opinion-preferred rewiring is superior to the other rules under low rewiring rates, and coexistence occurs under high rewiring rates. For unbalanced proportions, the best response rule for the minority/majority is unfixed, and this depends on the initial proportion and rewiring frequency. Furthermore, we find evolution processes for all competing cases belong to two categories. Evolution Category I shows an obvious correlation between opinion proportions and the density of discordant edges (connecting nodes with different opinions), and these trends can be effectively described by numerical approximations. However, for Evolution Category II, no such correlation exists for individuals or linking pairs, and an analysis of local structures reveals the emergence of large numbers of open triads with the same opinions, denoting group prevalence. This work broadens the understanding of opinion competition and inspires exploring group strategies employed in social dynamic systems.

## Introduction

Opinion dynamics concern the ways in which different opinions evolve in a population, especially the ways that consensus is reached or the coexistence of several opinions. Researchers use theoretical models to understand processes of election^1^, persuasion and negotiation^2^, cultural transmission^3^, rumour cascades^4,5^, etc.

Early studies in opinion dynamics focused on shifts in individual opinions occurring within a fixed topology. Opinions are characterized as discrete integers (voter model^6^, majority-rule model^7^, Sznajd model^8^, etc.), continuous real numbers (Deffuant model^9–11^, Hegselmann-Krause model^12^, etc.), or vector-represented features^13–16^ in diverse situations, and effects of herding^17,18^, homophily^14,19,20^ or social influence^21–23^ are embedded in the ways that opinions are updated. Studies were then extended to consider multiple dimensions, adaptation in network structures (i.e., preferential attachment^24,25^, rewiring^26–28^, and cut-offs^29^), and heterogeneities between individuals (e.g., opinion leaders^30,31^, extremists^9–11,32–34^, and delayed individual interactions^35–37^). Among these, the coevolution of dynamics of network topology and the interactions of individual opinions have been of particular concern^38–41^.

A paradigmatic coevolution opinion dynamic model has two parts: the opinion update mechanism and the structure update mechanism^26,28,42^. The voter model^1,6,43^ and Deffuant model^2,16^ are two typical opinion update mechanisms for modelling updates of discrete opinions or opinions of a bounded confidence range^16,21,44^. The opinion update mechanisms follow opinion models on a fixed topology. For the structure update mechanism, various rewiring behaviours (also known as partner-switching) have been considered due to their effects on network structures and opinion proportions, especially random rewiring^26,42^, homophily-based rewiring^14,19,20,24,45^, and, recently, rewiring based on local topologies, heterogeneities, or additional geographic information^25,46,47^. Therefore, a large body of research treats coevolutionary opinion dynamics as a spin system at the individual level^16,48^, and rewiring behaviours are considered unified for all individuals, while opinion dynamics occurring at the group level and inhomogeneous rewiring among groups have not been discussed.

For highly organized groups, such as political parties or religious systems, individuals in the same group share converged behaviours^3,49–52^, which are also called political or religious behaviours^13,50^. This is partly due to beliefs, affiliations, commitment rules and taboos^53^ that a party or religion applies to restrict its members to certain patterns of behaviour. Individuals who share the same opinions may act similarly to create a group identity or ingroup bias^45,54–56^. As an empirically blurry case, for some religions, missionaries more openly preach their beliefs (Christianity) even when they maintain designated places for activities (e.g., churches), while for other religions, religious practice is more restricted to certain areas (temples)^57^, and modes of spreading beliefs are more restrained (Buddhism). As another example, lovers with different beliefs face considerable pressures from religious rules and from their companions, causing such couples to be unstable and often leading to separation simply due to their beliefs in different gods^58–60^. Therefore, the ways in which group members adopt consistent behaviours during group expansion or group conflict form an objective and complex phenomenon^51,52,61,62^. As an abstract representation, when different opinion groups adopt different group strategies, i.e., more heavily biased to high-degree individuals or drawing closer to individuals with similar opinions, to spread their ideas, to absorb new believers or to stabilize existing believers, the network topology is reshaped under, i.e., multiple rewiring behaviours. To the best of our knowledge, the compounding effects of multiple rewiring for opinion dynamics on adaptive networks has not been addressed, and thus, the effects of these group rewiring strategies on group expansion remain an interesting issue to explore.

Group-based rewiring rules can vary widely, even those based on the same properties can lead to varying rewiring behaviours. From a review of works on individual-based rewiring rules, we find that rewiring with no preferences^26,28,42^, opinion and homophily-based rewiring^19,28,63^, and network structure-based rewiring^64,65^ are three distinct types of rewiring behaviour. Random rewiring with no preferences on network structures or opinions (Rule I) is the most widely studied and default form of rewiring^16^, and we consider it a benchmark. Rewiring to nodes with the same opinion as itself (Rule II) and with no limitations on other properties, such as network properties, represents a basic mode among homophily-based rewiring or opinion-based rewiring approaches, as other forms of homophily-based rewiring may take other factors into consideration^14^. Moreover, node degrees are key features of network structures, and preferentially rewiring to nodes with high degrees without considering opinions (Rule III) represents the essence of preferential attachment models^66,67^.

Here, we consider a case of binary opinion competition in which each opinion group adopts its own rewiring behaviours as a group strategy in a generalized coevolution voter model. This framework represents a pioneering attempt to embrace non-unique rewiring behaviours under a uniform evolutionary process. The extended model allows one to determine whether a superior rewiring rule exists under initially balanced opinion proportions and proper response rules to apply when given an opponent’s choice, in which the ordinary coevolution models do not support disentangling.

We find that the dynamic processes of various competing cases involve two forms of evolution: one involves mapping correlations between opinion proportions and the density of discordant edges, while the other does not. For evolution of the first category, we provide a mathematical description of the evolutionary process (including the final competition results) with simulation and numerical approximations. For evolution of the second category, we analyse local structures, especially open triads with opinions, and we address their role in group shares.

## Methods

### Adaptive voter model with multiple rewiring rules

Our model is based on social influence and group behaviour (SIGB): social influence is modelled by an adaptive voter model, while a pre-adopted rewiring rule serves as a proxy for group behaviour. The SIGB model involves four steps. (1) Pre-sets: initially, the network contains *N* nodes {*v*_1_, *v*_2_, …, *v*_*n*_} and *M* = *kN* undirected random edges (Erdös-Rényi graphs). Each node *v*_*i*_ employs one of two opinions *o*_*i*_ called 0 and 1; opinion 1 has an initial proportion of $a\in (0,1)$, and opinion 0 has an initial proportion of $\,1-a$. Two rewiring rules are bound to the two opinion groups and fixed; i.e., nodes with opinion 0 adopt one rule, and nodes with opinion 1 adopt the other rule. (2) At each time step *t*, a discordant edge *e*_*ij*_ with opposing opinions on each side is selected randomly. (3) With probability 1-*w*, one node *v*_*i*_ imitates the opinion of the other node *v*_*j*_; otherwise, with probability *w*, the link between them is broken, and one of them forges a new connection to another node following the rewiring rule that its opinion group adopts. (4) This process stops when no discordant edges connect nodes to different opinions any longer. The SIGB model reflects an integration of Durrett’s adaptive voter model^28^, but one or two rewiring rules can coexist in one dynamic process. Therefore, the SIGB model can degenerate to uniform rewiring behaviours at the individual level (i.e., when both opinion groups adopt the same rewiring rules) as in previous coevolution models, and it can update to rewiring behaviours at the group level and advance the understanding of effects of multiple rewiring rules on opinion group competition. We note that the rewiring rate *w* is the same for all individuals; we also hypothesize that individuals first admit these new links and then decide whether to update opinions or social relations. We do not take the refusal of rewired links into consideration. This is a strong hypothesis.

**Figure 1:**
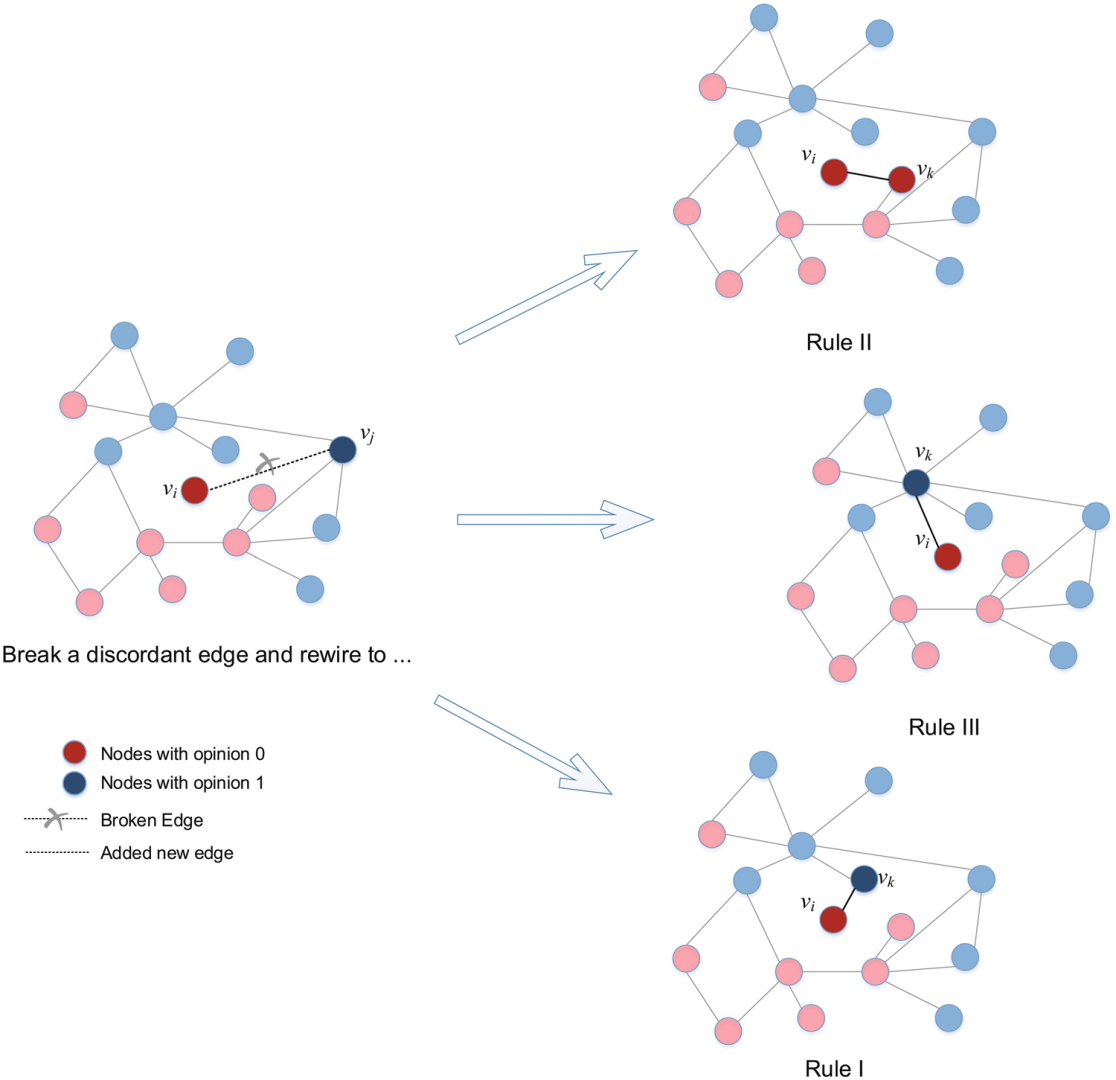
Rewiring under different rules.

Group-based rewiring rules can be diverse, can be based on the same properties and can lead to varying rewiring behaviours. From a review of rewiring rule-related works, we find that random rewiring (with no preferences on network structures or opinions, Rule I), opinion-preferred rewiring (rewiring to nodes with the same opinion, Rule II), and degree-preferred rewiring (preferentially rewiring to nodes with high degrees without consideration of opinions, Rule III) are three typical and distinct forms of rewiring behaviour. Rule I applies no preferences, i.e., one node randomly rewires to another node (not to itself or its neighbour) at the global network scale. Rule II forges an opinion-preferred attachment. Rule III involves a degree-preferred attachment. Under Rule III, the current node tends to rewire to another node *ν* with degree ${D}_{v}$ and probability $p={D}_{v}/\sum _{{v}_{i}\in U}{D}_{{v}_{i}}$, where *U* is the node set of all nodes (see Fig. 1). These three rewiring rules can be well-expressed during the coevolution of network structure adjustments and node opinion updates, and they represent abstract and basic approaches that can be combined or varied to ensure more complex rewiring behaviours. Table 1 presents a feature-represented comparison of the three rewiring rules. For binary opinion competition dynamics, each opinion group applies its own rewiring rule for 9 (3 × 3) competitive cases. In fact, this vector-like representation is highly scalable for future studies on other rewiring rules, and any rule with *k* features can be characterized as a 1 × *k* vector, as shown in Table 1.

**Table 1:** Feature-represented rewiring rules with preferences for opinions and structures.

Rewiring rules Preference	Rule I Random	Rule II Opinion-preferred	Rule III Degree-preferred
Preference for opinions	None	Same opinion	None
Preference for structures	None	None	High degree (probability)

According to Durrett’s model^28,68^, when a consensus exists, the expected time can be reached in ${\rm O}(N\,\mathrm{log}\,N)$ steps when $w=1.0$ and in ${\rm O}({N}^{2})$ steps when $\,w=0.0$ (any power between 1 and 2 generates the same results when $N\to \infty$). Evolution would finally stop under a finite population. As our SIGB model is similar to Durrett’s, this finite evolution period remains constant. From the following results, we measure changes in opinion proportions from final to initial shares, and the final proportions of opinions are used to determine superior rewiring rules.

## Results of the Binary Opinion Competition

We first examine a special case in which two opinion groups share a balanced initial proportion ($a=0.5$). In this case, neither group rewiring strategy presents a 100% probability of generating a larger final share, but one group strategy may present a clearly larger probability of doing so; we call this the “superior rewiring rule” under a balanced initial proportion. We then examine a general case that removes strict rules on unbalanced initial opinion shares ($a\in (0,0.5){\cup }^{}(0.5,1)$). Our main interest is to identify rewiring rules that promote group size expansion and win the competition.

### Superior rewiring rules under a balanced initial proportion

Under a balanced initial proportion, the initial share for both opinion groups is 50%, while the final share is non-deterministic: sometimes opinion 0 wins, sometimes opinion 1 wins more, and sometimes their final difference is insignificant (e.g., 0.1%), in which case it is inaccurate to say that one rewiring strategy is guaranteed to win over the other. To capture changes occurring in the final opinion proportion over the initial share, a conversion ratio, *r*, is introduced (only to the balanced initial proportion).

1 $$r=|\frac{{\rm{Final}}\_{\rm{Proportion}}-{\rm{Initial}}\_{\rm{Proportion}}}{{\rm{Initial}}\_{\rm{Proportion}}}|$$

When the density of 1 s increases to greater than *r*, opinion 1 dominates, and when the density of 0 s increases to greater than *r*, the opposite occurs. When neither opinion promotes *r*, they are regarded as being in coexistence. A special case occurs when *r* = 0%, as coexistence is excluded, and the competition results would then be that one always dominates and the other loses. As a demonstration, we fix $r=10 \%$. We also test for cases *r* = 5%, 10%, 15%, and 20%, which do not change the conclusion presented here. By simulating 300 times, we record the change in opinion 1 wins, opinion 0 wins and coexistence, and we use a ratio of the main trend (RMT) to determine the three proportion values.

**Figure 2:**
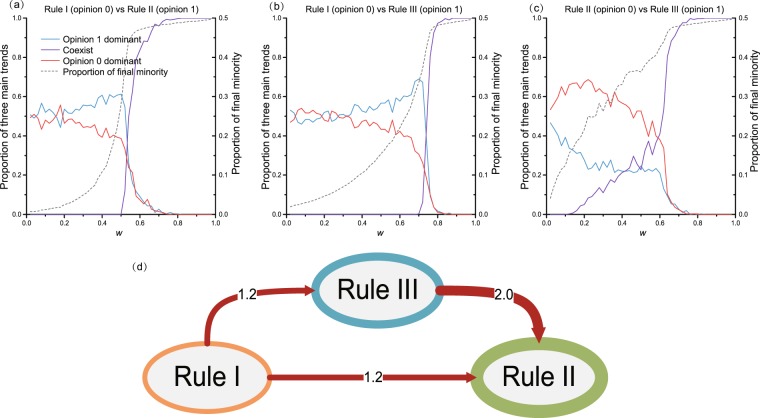
Binary opinion competition with a balanced initial proportion. (**a–c**) Opinion competitions where *a* = 0.5 under non-unique rewiring rules. (**a**) Rule I (opinion 0) vs. Rule II (opinion 1). The left-hand y-axis denotes the proportions of three main trends: opinion 1 dominant (blue line), opinion 0 dominant (red line), and coexistence (purple line) under the rewiring rate *w*. The right-hand y-axis denotes the proportion of the final minority under *w* (grey dotted line). (**b**) Rule I vs. Rule III. (**c**) Rule II vs. Rule III. (**d**) Accumulated advantage ratio for rewiring rules. For example, for Rule I vs. Rule II, the accumulated trend for opinion 1 being dominant (Rule II wins) is 1.2 times the value of the reverse trend. Here, *N* = 2000, and *M* = 2 *N*.

The results for the case when both opinion groups adopt the same rewiring rule are listed in Appendix A, Fig. S1 (see also ref.^28^ Figs 1 and 2). A comparison of these results shows that adopting non-unique rewiring rules breaks the balance and causes a favouring of one opinion. For Rule I (adopted by individuals of opinion 0) versus Rules II or III (adopted by individuals of opinion 1), under low rewiring rates (*w* < 0.2), the two opinions cannot coexist, i.e., either opinion group gains a comparative advantage over the other, and this slight advantage finally turns to dominance (see Fig. 2(a,b)). Under moderately frequent rewiring levels (i.e., *w* = 0.5), the proportion of opinion 0 s with Rule I declines, and its disadvantages are clearly enhanced (see Fig. 2(a,b)). For Rule II vs. Rule III (see Fig. 2(c)), even under a low rewiring rate *w* = 0.2, Rule II suppresses Rule III, and the resulting significant advantage persists until the rewiring rate increases to *w* = 0.6. In contrast to this low rewiring rate, when *w* is very high ($w\ge 0.8$), the two pairs of games (Rule I vs. Rule II, Rule I vs. Rule III, Rule II vs. Rule III, and Rules vs. themselves, with $a=0.5$) lead to coexistence, and the difference observed is the specific value of *w* that reaches this coexistence stage (see Fig. 2(a–c) and Appendix A, Fig. S1). The auxiliary line for the proportion of the final minority denotes the critical point of *w* at which coexistence is reached. Therefore, no group rewiring rule can 100% ensure the dominance of the opinion group adopting it, and for different rewiring rates, competition results can be captured from the probability of three main trends under a balanced initial proportion. We ran 300 simulations and recorded the final proportion of opinions for $w\in (0,1)$ with *N* = 2000 nodes and an average degree of 2*k* = 4.

Based on these, we calculated a cumulative advantage ratio (CAA) for each competing pair over the range of rewiring rates. For Rule *x* vs. Rule *y* (*x* and *y* can be I, II, or III), we calculate the cumulated RMT for opinion groups adopting Rule *x* versus the cumulated RMT for those adopting Rule *y*:

2 $$CA{A}_{y\to x}=\frac{{\int }_{0}^{1}RM{T}_{x}(w){\rm{d}}w}{{\int }_{0}^{1}RM{T}_{y}(w){\rm{d}}w}$$

where *RMT*_*x*_(*w*) is the proportion of trends that groups adopting Rule *x* dominate. The cumulated value provides an evaluation without considering *w*. For example, $CA{A}_{{\rm{III}}\to {\rm{II}}}$ = 2.0 (edge from Rule III to Rule II) representing the cumulative RMT for the group adopting Rule II is twice the cumulative advantage of Rule III and vice visa (see Fig. 2(d)). The same applies for the other two edge weights. *CAA*s of opinion competition under rewiring rules obey monotonous win and loss results: Rule II > Rule III > Rule I. Thus, under an initial balanced proportion, even when the rewiring rate is not given, Rule II denotes as a preferred strategy for opinion groups.

Superior rules for the initial minority/majority under an unbalanced initial proportion

**Figure 3:**
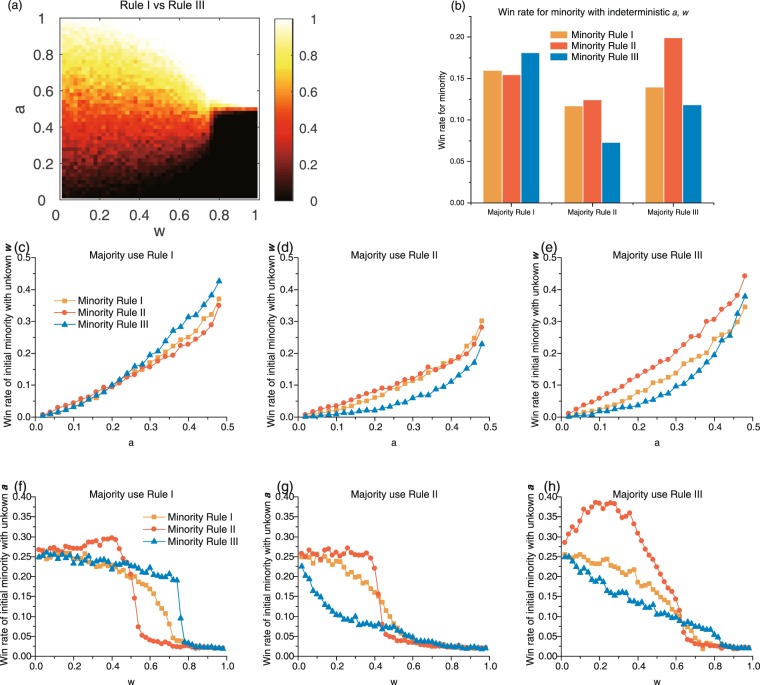
Responses of the group holding opinion 1 under various conditions. (**a**) The win rate of the group holding opinion 1 s under various *w* and *a* values for Rule I vs. Rule III. The other competing pairs are given in Appendix B, Fig. S2. For figures (**b–h**), we have *a* < 0.5, so opinion 1 holders form the initial minority. (**b**) Win rates for the minority with indeterministic *a* and *w* values. This is the average win rate for $w\in (0,1)$ and $a\in (0,0.5)$. (**c–e**) Win rates for the minority with a given majority and *a* but with indeterministic *w*. Orange dots denote the minority adopting Rule I, red dots denote the minority adopting Rule II, and blue dots denote the minority adopting Rule III. (**c**) The majority adopts Rule I. (**d**) The majority adopts Rule II. (**e**) The majority adopts Rule III. (**f–h**) The win rate of the minority for a given majority and *w* and for indeterministic *a*. (**f**) The majority adopts Rule I. (**g**) The majority adopts Rule II. (**h**) The majority adopts Rule III.

The unbalanced initial proportion considered is a more general case. For this case, we do not consider the state of coexistence, and the judgement of a winner is regarded as the final majority; i.e., when either side achieves more than a 50% final proportion, it wins. The probability of winning (win rate) for opinion 1 under Rule I (opinion 0) vs. Rule III (opinion 1) under $w\in (0,1)$ and $a\in (0,1)$ is given as an example in Fig. 3(a), and a panoramic view of all competing cases (three cases for one rewiring rule adopted by both opinion groups and three cases for two different rewiring rules adopted) is shown in Appendix B, Fig. S2. For a given *w*, the initial proportion of opinion 1 is strongly correlated with its final proportion, and the win rate is asymmetric at $a < 0.5$ and $a > 0.5$, as the two rewiring rules are different. When opinion 1 is the initial minority ($a < 0.5$), a higher *w* value leads to the extinction of opinion 1 ($a\approx 0$), and when opinion 1 forms the initial majority ($a > 0.5$), a higher *w* leads to a consensus on opinion 1 ($a\approx 1.0$). A higher rewiring rate denotes the occurrence of more updates on a network structure and fewer updates on individual opinions, meaning that individuals prefer to change social relations rather than change their or their neighbours’ opinions. This creates an ingroup bias and divides the graph into two main sub-components, with each including individuals sharing a consensus and divided components holding different opinions. As the network structure is severely divided, the initial minority is presented with fewer opportunities to influence individuals supporting the majority, and thus, their chance of winning drops to nearly 0%. However, when the rewiring rate is low, effects of social influence support the formation of one large and connected component, and thus, the initial minority can influence more individuals, overtake the majority and not be limited by the initial share. Therefore, the initial proportion is positively correlated with the final share, and a high rewiring rate amplifies the impact while suppressing effects of the rewiring rule that the initial minority adopts.

The results shown in Figs 3(a) and S2 offer a perfect perspective of deterministic *w* and *a*, and we further consider the cumulated win rate of *w*, *a* or both, which can be seen as a game with incomplete information. In such situations, the opposite strategy is employed, and some parameters (*w* or *a*) are indeterministic (i.e., both *w* and *a* are not given, or only *w* is given) in the a priori proper selection of the rewiring rule for the minority (assuming a uniform distribution of *w* and *a* in the whole range). Figure 3(b) presents the win rate of minority ${{\rm{WR}}}_{Min}$ under an indeterministic *w* and *a* (denoted as $\bar{w}\,{\rm{and}}\,\bar{a}$):

3 $${{\rm{WR}}}_{Min}(\bar{w},\bar{a})\approx \frac{{\iint }_{F}{{\rm{WR}}}_{Min}(w,a){\rm{d}}a\,{\rm{d}}w}{{\iint }_{F}{\rm{d}}a\,{\rm{d}}w}$$

with $F:0 < w < 1,\,0 < a < 0.5$. When the initial majority adopts Rule I, the a priori adoption of a group strategy by the minority is Rule III; similarly, the a priori choice of the minority when the majority uses Rule III is Rule II, and when the majority adopts Rule II, the a priori choice made by the minority is either Rule II or Rule I (their win rates are very similar), with Rule II being slightly better.

Similarly, Fig. 3(c–e) show win rates of the minority under an indeterministic rewiring rate *w* given the opponent’s strategy and initial proportion *a*:

4 $${{\rm{W}}{\rm{R}}}_{Min}(\bar{w},a)={\int }_{w=0}^{1}{{\rm{W}}{\rm{R}}}_{Min}(w,a)\,{\rm{d}}w/{\int }_{w=0}^{1}{\rm{d}}w$$

In these cases, as initial proportion *a* increases, ${{\rm{WR}}}_{Min}(\bar{w},a)$ increases. The results for the a priori choices of the minority (Fig. 3(c–e)) are consistent with the results shown in Fig. 3(b), but in some cases, the best response choice can be different. When the majority adopts Rule I (Fig. 3(c)), when *a* < 0.2, all three choices made by the minority are close on ${{\rm{WR}}}_{Min}(\bar{w},a)$; only when *a* > 0.2 does Rule III present an advantage over the other two (Fig. 3(c)). When the majority adopts Rule II (Fig. 3(d)), either choice made by the minority presents a lower win rate than when the majority adopts other rules. Even when $a\in [0.45,\,0.48)$, ${{\rm{WR}}}_{Min}(\bar{w},a)$ is still less than 30%, reflecting the dominance of Rule II when adopted by the majority. When the majority adopts Rule III (Fig. 3(e)), the best response rule for the minority is always Rule II, revealing its advantages over the whole range of *a*. Thus, effects of rewiring rules show different sensitivities towards the initial proportion, and when the majority adopts Rule I or Rule II, the best response choice for the minority relates to the initial proportion *a*; when the majority adopts Rule III, the best response choice for the minority is always Rule II.

Figure 3(f–h) show win rates for the minority under an indeterministic initial proportion *a*:

5 $${{\rm{WR}}}_{Min}(w,\,\bar{a})={\int }_{a=0}^{0.5}{{\rm{WR}}}_{Min}(w,a)\,{\rm{d}}a/\,{\int }_{a=0}^{0.5}{\rm{d}}a$$

${{\rm{WR}}}_{Min}(w,\,\bar{a})$ always decreases under a high rewiring rate (*w* > 0.6), as high structure and low opinion updates initial suppress a minority takeover. Under a low *w*, the win rate of the minority shows diverse patterns. In a typical case, the win rate of the minority first undergoes a gentle decline and then declines rapidly as *w* increases. This can be observed when the majority adopts Rule I and the minority adopts all three choices (Fig. 3(f)), when the majority adopts Rule II and the minority adopts Rule I or II (Fig. 3(g)) and when the majority adopts Rule III and the minority adopts Rule I (Fig. 3(h)). In another typical case, the win rate of the minority always decreases as *w* increases. This occurs when the majority adopts Rule II, when the minority adopts Rule III (Fig. 3(g)) and the majority adopts Rule III, and when the minority adopts Rule III (Fig. 3(h)). A special case occurs when the majority adopts Rule III and the minority adopts Rule II (Fig. 3(h)), in which the win rate of the minority first increases and then decreases as *w* increases. This reveals the advantages of Rule II over Rule III.

Rule II is the only rule that ensures that a majority minimizes its exposure to effects of the minority. When the minority does not also adopt Rule II, this implies that more members of the minority are influenced by majority members than the other way around. Even when the minority also adopts Rule II, this group still represents the minority and is thus more exposed to effects of the outgroup than the other way around.

The a priori response for the initial majority under a deterministic opposite choice and under an indeterministic *w* or *a* is shown in Appendix B, Fig. S3 based on the same method.

## Two Forms of Evolution Observed in Binary Opinion Competitions

### Two evolution processes

We further investigate the dynamic coevolution process and find explanations for competition results. One aspect concerns the connection between changes in opinion proportions and characteristics of network topology. For heuristic detection, we run the evolution of each competition several times and record at each time step the density of opinion 1 (${N}_{1}/N$) and the density of the minority opinion at the corresponding moment. We also record the density of 1–1 (${N}_{11}/M$) and 0–1 (discordant, ${N}_{01}/M$) edges.

**Figure 4:**
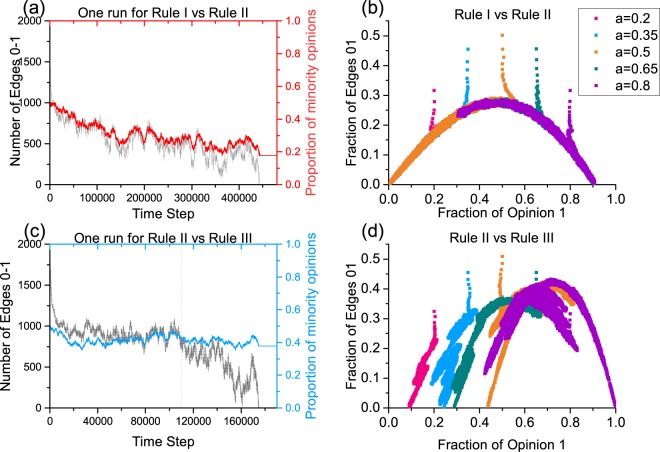
Two categories of the evolution process. (**a**) Example of evolution in Category I (Rule I vs. Rule II). The black line denote the number of 0–1 edges observed per time step. The red line denotes the proportion of minority opinions involved. (**b**) Example of evolution in Category II (Rule II vs. Rule III). The black line denotes the number of 0–1 edges per time step. The blue line denotes the proportion of minority opinions. The two evolution processes illustrated in (**a**,**b**) are run once under *w* = 0.4 and *a* = 0.5. (**c**) Observed arches for Rule I vs. Rule II (opinion 1). (**d**) The fraction of 0–1 edges vs. the fraction of 1 s for Rule II vs. Rule III (opinion 1). Both (**c**,**d**) are run under *w* = 0.4 and *a* = 0.2, 0.35, 0.5, 0.65, and 0.8.

We find that all six cases of competing pairs belong to two types of dynamic processes. As the key phenomenon in evolution Category I, after an initial decrease in discordant edges, there is a strong correlation between the proportion of the minority opinion and the number of 0–1 edges. This correlation then persists until the end of the evolution period (see Fig. 4(a)). At the end of the evolution period, the number of 0–1 edges drops to 0, and the density of the minority also decreases to a certain value (roughly 0.2). The difference in evolution Category II is that, at the end of evolution, the decreasing number of 0–1 edges shows no such correlation with the density of the minority opinion; when the number of 0–1 edges presents a larger amplitude and drops to 0.0, the density of the minority remains relatively high and only changes slightly (roughly 0.4, see Fig. 4(b)). Rule I (opinion 0) vs. Rule II (opinion 1) and Rule II (opinion 0) vs. Rule III (opinion 1) serve as examples of the two evolution categories. Both were run one time for the whole evolution process with *N* = 2000, *M* = 4000, *w* = 0.4, and *a* = 0.5. These phenomena are always present, though the results of evolution Category II can vary. All evolution categories of the competing pairs are shown in Table 2.

**Table 2:** Group competing pairs and their evolution categories.

	Category I	Category II
Symmetric cases	Rule I vs. Rule I Rule II vs. Rule II	Rule III vs. Rule III
Asymmetric cases	Rule I vs. Rule II Rule I vs. Rule III	Rule II vs. Rule III

The initial transition and relationship can be observed more clearly from a cumulative scatter view of pairs $({N}_{1}/N,{N}_{01}/M)$ for different values of *a*. For Category I, after the initial transition, different evolutions initiate from *a* = 0.2, 0.35, 0.5, 0.65, and 0.8, and all share a unified arch-like relationship between the density of 0–1 edges and the density of opinion 1 (see Fig. 4(c)). Rather, after the initial transition, the density of 0–1 edges can be regarded as a function of a given density for opinion 1. The arch-like evolution process ends when ${N}_{01}=0$ with roots ${\theta }_{{c}_{1}}\in [0,0.5)$ and ${\theta }_{{c}_{2}}\in (0.5,1.0)$, which are the two possible final group shares for evolution starting with $a\in [{\theta }_{c1},{\theta }_{c2}]$ bound on the arch. When $a\in [0,\,{\theta }_{{{\rm{c}}}_{1}})\cup ({\theta }_{{{\rm{c}}}_{2}},1]$, evolution quickly converges, and the final density is very similar to its initial share with a slight shift towards 0.0 or 1.0. The difference for evolutions in Category I is the specific features of the arch-like relationship. For Category II, there is no such unified arch-like correlation (see Fig. 4(d)), and the final density is dependent on the initial share *a*. Thus, evolutions in Category II involve more complex phenomena than those in Category I.

For evolutions in Category I, an arch-like correlation between the density of opinions and the density of discordant edges forms when either opinion group adopts Rule I (Rule I vs. Rule I or II or III) or when both use Rule II (Rule II vs. Rule II). All of these cases involve a Poisson-like degree distribution. Symmetric arches have been discussed by Cox JT and Greven^69^ and Durrett^28^ for the Rule I vs. Rule I case and by Vazquez *et al*.^63^ and Durrett^28^ for the Rule II vs. Rule II case. However, asymmetric cases involving Rule I vs. Rule II and Rule I vs. Rule III have not yet been addressed, and for evolutions in Category II, i.e., opinion competitions under Rule II vs. Rule III and under Rule III vs. Rule III, an arch-like correlation is not observed, especially for the final case. These patterns of evolution remain less understood.

Therefore, in the following parts of this section, we separately analyse these two forms of evolution.

### Category I: Evolution satisfying a quasi-stationary distribution

#### Two absorbing states and competition results

Here, we present a more general and asymmetric case involving Rule I vs. Rule II, and we then explore other patterns of evolution in Category I.

**Figure 5:**
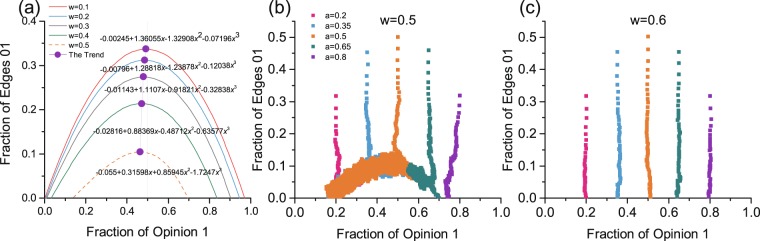
Evolution for competing cases for Rule I vs. Rule II. (**a**) Observed arches for Rule I vs. Rule II with *w* = 0.1, 0.2, 0.3, 0.4, and 0.5. The purple line denotes the trend of maximum points on these arches. (**b**) Evolution when *w* = 0.5. (**c**) Evolution when *w* = 0.6. The specified parabolas fit the simulation data with *N* = 2000 and *M* = 4000.

Figure 4(c) presents opinion competition results generated for Rule I vs. Rule II with rewiring rate *w* = 0.4. In Fig. 5, we consider other rewiring rate and present curves for the observed arches. Under a sequence of rewiring rates $w=0.1,0.2,0.3,0.4$, the maximum point ${Z}_{w}$ on the arch with coordinate states $\,({Z}_{{w}_{1}},{Z}_{{w}_{01}})$ always meets ${Z}_{{w}_{1}} < 0.5$, and coordinate states $({Z}_{{w}_{1}},{Z}_{{w}_{01}})$ shift to smaller values ${Z}_{{w}_{1}}$ and ${Z}_{{w}_{01}}$ as the rewiring rate *w* increases. The right absorbing root ${\theta }_{{{\rm{c}}}_{2}}$ shifts away from 1.0, and the left absorbing root ${\theta }_{{c}_{1}}$ remains close to 0. Thus, for evolution bound on the arch ($a\in [{\theta }_{{{\rm{c}}}_{1}},{\theta }_{{{\rm{c}}}_{2}}]$), maximum points on the arch present a bias at ${Z}_{{w}_{1}} < 0.5$; as rewiring rate *w* increases, the range of $({Z}_{{w}_{1}},1]$ increases, and competition results are biased to more opinion 1 holders. Therefore, Rule II (adopted by opinion 1) is superior to Rule I. The specified arches are fits to simulation results of *N* = 2000 and *M* = 4000. The phenomena described above also apply to the case of Rule I vs. Rule III (see Appendix C, Fig. S4).

**Figure 6:**
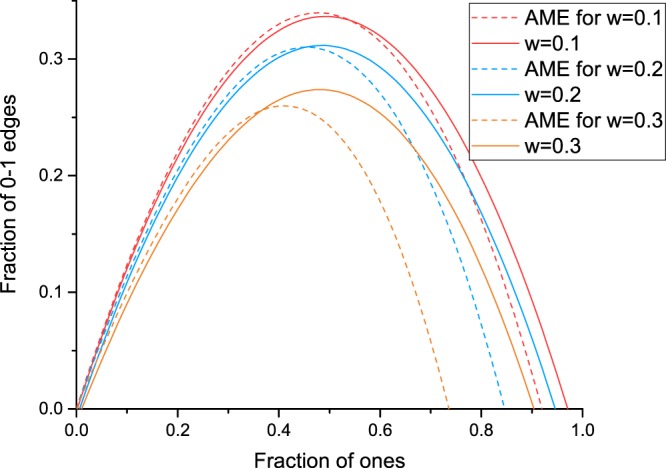
Arches computed from an approximate master equation (AME) versus simulation under Rule I vs. Rule II with *w* = 0.1 (red), 0.2 (blue), and 0.3 (orange). The solid line shows solved and fitted results derived from the AME, while the dotted line shows the simulation results.

These asymmetric arch-like curves essentially differ from those for which both groups adopt the same rewiring rules (all individuals adopt the same rewiring rules), where maximum points on the symmetric arch never shift away from ${Z}_{{w}_{1}}=0.5$, as noted in Refs^28,63,69^. When all individuals adopt the same rewiring rule and the initial proportion is 0.5, both opinion groups have an equal chance of winning, and their competition would always have a winner in the end (see ref.^28^ Figs 4, 6 and 8).

The fact that the arch-like correlation identified after the initial transient, ${N}_{10}/M$, can be regarded as a function of ${N}_{1}/N$ is a sign for evolutions in Category I. Similar phenomena can be observed from the Wright-Fisher diffusion process and from Durrett’s twin models, which support the assumption that the evolving voter model includes a one-parameter family of quasi-stationary distributions^70^. That is, the values of all graph statistics can be computed from ${N}_{1}/N$, including the final opinion proportion. The difference lies in the fact that the Wright-Fisher process and Durrett’s models only apply to symmetric arches, which only provide partial descriptions of evolution occurring under Rule I vs. Rule I and under Rule II vs. Rule II and cannot provide a full description of all cases of Category I. Therefore, we provide extended formulas that include the asymmetric competing cases of Rule I vs. Rule II and of Rule I vs. Rule III as well as the symmetric ones. Further information on evolutions in Category I is given in Appendix D.

Evolutions in Category I presents a nontrivial correlation of trends in opinion proportion and network structure characteristics. It is natural to assume that these processes can be described using mathematical tools, especially via statistics and approximate calculus. The approximate master equation (AME) framework^71,72^ has been proven to be better at estimating *ρ* than mean-field equations or pair approximates in voter models, epidemic models, and other binary state dynamics, and it is applied to competing symmetric cases^28^. In the next subsection, we apply a modified AME for all evolutions in Category I.

#### Approximate calculations on coevolution

The AME framework considers three types of events: (i) rewiring may break the link between node *x* and *y* and create a new edge connected to *x*; (ii) *x* or *y* may influence the other by a voting step; or (iii) the opinion of *y* may be changed by imitating one of its neighbours $z\ne x$. Suppose that ${S}_{k,m}(t)$ is the number of nodes measured at time *t* that are in state 0 and that ${I}_{k,m}(t)$ is the number of nodes in state 1, both having degree *k* and *m* neighbours in state 1. Exact equations can be written for the first two types of events in terms of ${\bar{S}}_{k,m}$ and ${\bar{I}}_{k,m}$, and the third type involves making an approximation. In solving the equations, the AME provides the estimated results of opinions 1 and 0 for each time step. The detailed methods of the AME framework are given in Appendix E.

The conventional AME can describe the evolutionary process under the symmetric rewiring of Rule I vs. Rule I and of Rule II vs. Rule II^28^. However, for asymmetric cases, such as Rule I vs. Rule II, we find that it can only describe the initial transient stage well (see Fig. S5(a)) and is limited in describing the following process. This can be attributed to the bias of using infinite population cases to approximate finite populations with heterogeneous network topologies.

Our estimations made here for asymmetric cases are based on the critical stage calculated from the AME between the initial transition and the following dynamic process. By fitting states $({N}_{1},\,{N}_{01})$ at critical stages under different *a*, we have the approximate evolution result (see Fig. S5(b)). Further information on the identification of the critical stage is given in Appendix E. The approximation generated by the AME is shown in Fig. 6 and involves arch-like phenomena, as shown in Figs 4(c) and 5. As *w* increases, the arch-like curve collapses, the right root shifts away from coordinate (1.0, 0.0), and the maximum points shift to smaller $({Z}_{{w}_{1}},{Z}_{{w}_{01}})$ values. The AME provides an estimate of qualitative behaviour: the predicted $\rho (a) > 0$ for all $a\in (0,1)$ and tends to 0 as $a\to 0$. Thus, the evolution process of Rule I vs. Rule II is effectively illustrated via approximate calculus, and competition results of winning or losing are consistent among simulations and approximations. One can repeat the analysis described above for the case of Rule I vs. Rule III, and corresponding details are given in Appendix E.

### Category II: No quasi-stationary distribution occurs during evolution

Unlike evolutions in Category I, opinion competitions under Rule II vs. Rule III and Rule III vs. Rule III do not maintain an arch-like correlation of opinion proportions and discordant edges on *a*, especially in the final stage of evolution. Thus, evolutions in Category II do not involve the one-parameter family of a quasi-stationary distribution, and the existing AME framework is not applicable to these evolution cases. In addition, the opinion proportion is determined at an individual level, discordant edges are determined at the level of linked pairs, and these levels show limitations for understanding the evolution. In particular, the temporary majority may not be the final winner. These obstacles force us to examine a deeper level of substructures or local structures in the network, such as closed triads or open triads (two edges linking three nodes). The quantity gap between open triads and closed triads here is 2–3 orders of magnitude; in addition, the number of closed triads can result in misjudging the advantaged opinion group.

**Figure 7:**
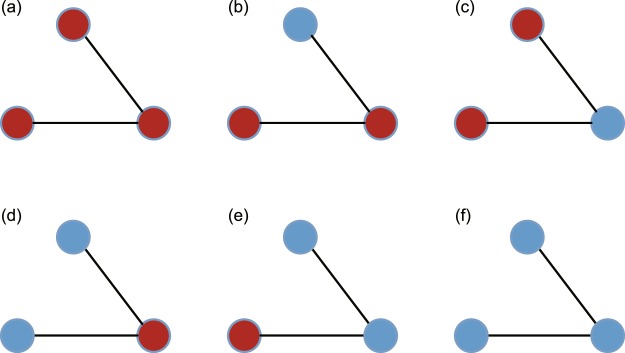
All possible open triads with opinions. Red nodes denote opinion 0, and blue nodes denote opinion 1.

As nodes have binary opinion states, the total number of open triads is measured as 6 (see Fig. 7). The number of open triads can be affected by three factors: (i) The initial number of opinion holders: The initial majority opinion correlates with a larger number of open triads, in which 2 or 3 nodes in an open triad may hold opinions that respond to the initial majority. (ii) Opinion updating processes: During opinion updating processes, open triads with different opinions (Types b-e in Fig. 7) are more likely to transmit (we refer to these as unstable open triads), while in the final stage, only triads with the same opinions (Type a and f in Fig. 7) remain (we refer to these as stable open triads). (iii) Rewiring processes: Different rewiring behaviours perform different functions in adjustments of network topology. Rule I shows no obvious preferences in terms of forming triads, but it can connect different components and can be regarded as a weak tie mechanism. Rule II shows a preference for forming open triads with the same opinions, which are also the two types that remain stable at the end of evolution. Rule III prefers to form giant connected blocks in which several nodes present extremely high degrees, and it shows no preferences for certain types of triads. Stable open triads can endure while unstable open triads are decomposed throughout the process and finally disappear. The number of triads during and after the evolution may play a role in the final proportion of corresponding opinions present.

**Figure 8:**
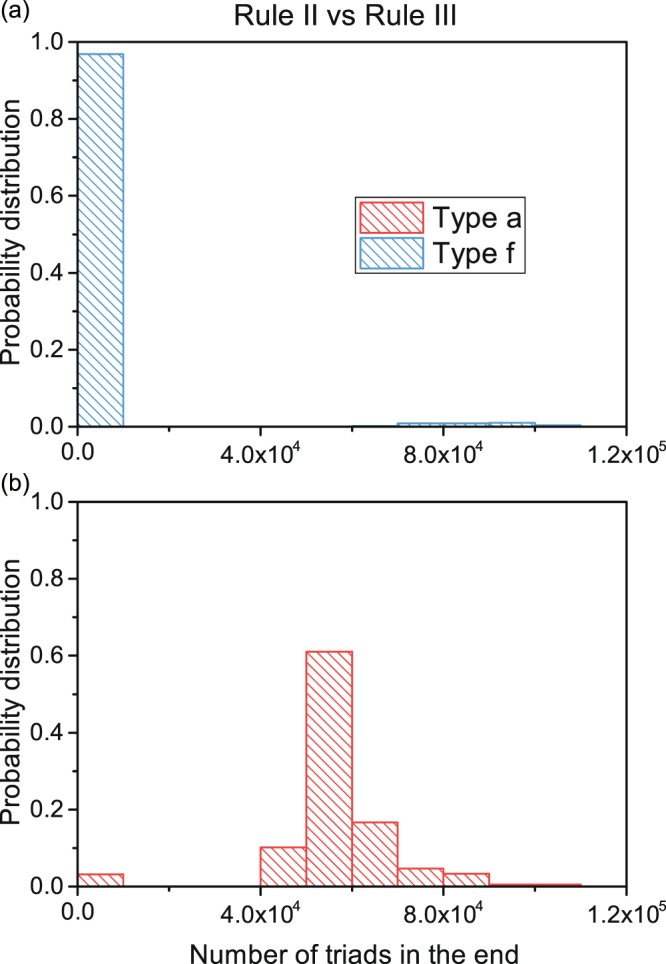
Final number of two triads after 500 simulations. (**a**) Bar chart for the final number of Type f triads for all simulation results. (**b**) Bar chart for the final number of Type a triads for all simulation results. Blue means that the open triads contain three opinion 1 s, and red means that the triads contain three opinion 0 s. The histogram shows the number of triads and the ratio of numbers. Over 95% of all blue triads fall within the range of (0, 10^4^), and over 95% of red triads fall within the range of (4 × 10^4^, 9 × 10^4^).

We conduct a case study on Rule II vs. Rule III. We run simulations 500 times under balanced initial proportions (with *w* = 0.3) and record the distribution for a final number of two stable open triads (see Fig. 8). The number of Type a and Type f opinion triads is very similar in the initial stage under a randomly generated network. However, in the final distribution, open triads with all 0 s showed a much larger deviation and remained in large numbers, while triads with all 1 s shrank to small amounts with high probability. Therefore, remaining stable open triads (possibly more triads of Type a for 0 s) could serve as an indicator of competition results, i.e., the opinion group applying Rule II wins with a higher probability than that applying Rule III. This is attributable to the different preferences for forming open triads in the rewiring rules, and this conclusion applies to all competing cases.

**Figure 9:**
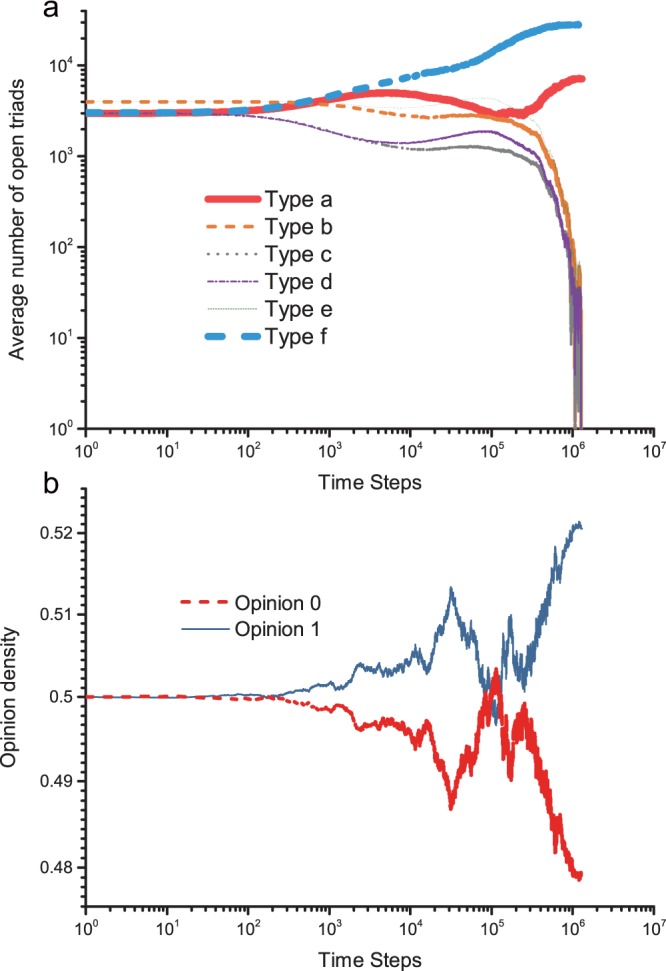
Number of evolving triads and changes in opinion density per time step. (**a**) The number of evolving triads per time step. The red line applies to triad Type *a*, the orange line applies to Type *b*, the grey line applies to Type *c*, the purple line applies to Type *d*, the green line applies to Type *e*, and the blue line applies to Type f. (**b**) The average opinion density per time step. Opinion 0 is shown as a red line, and opinion 1 is shown as a blue line. Here, opinion 0 adopts Rule III, and opinion 1 adopts Rule II, *w* = 0.4, and *a* = 0.5. Node number *N* = 2000, and number of simulations = 300.

We further present a longitudinal analysis of the number of open triads relative to the density of opinion holders by time step in Fig. 9. The number of symmetric open triads (Type a and Type f, Type *b* and *e*, Type *c* and *d*) is initially similar at roughly 5,000 time steps (less than 1% of the whole evolutionary process), and the amount of Type f is more than 40% greater than that of Type a. However, at the 10,000~70,000 time steps, the density of opinion 1 is temporarily larger than the density of opinion 0 on several occasions, and the number of triads of Type a is always greater than (roughly 1.5 times to 2 times) that of Type f. In the end, opinion 0 adopting Rule II wins more frequently.

Therefore, the distribution of final amounts of stable open triads and the average number of triads during evolution show that it is the emergence of stable open triads in large numbers that is clearly associated over time with final opinion proportions. A gap in the number of stable open triads could indicate competition results even in early stages of evolution.

## Discussion

This work introduces the SIGB model, which considers opinion group-based rewiring rules applied within a generalized adaptive voter model. We examine three basic forms of rewiring (opinion-preferred rewiring, degree-preferred rewiring, and random rewiring) as alternative group strategies applied within the SIGB model. We compare binary opinion competition in both balanced and unbalanced initial opinion proportions and find rewiring rules promoting group expansion under deterministic or indeterministic initial proportions or rewiring rates. Further, we classify all dynamic processes of competing cases into two categories, from which we provide a mathematical description of evolutions in the first category with numerical approximations and simulations. We in turn find that the emergence of open triads with the same opinions in large numbers is a good indicator of the competition results of evolutions in the second category. Our results suggest that binary opinion competitions based on group rewiring behaviours can be effectively perceived in advance.

Although, as noted above, several works have been conducted on rewiring rules of opinion dynamics, our approach represents a pioneering attempt to broaden research conducted at the individual level to the opinion group level and to convey diverse rewiring rules as group strategies that reflect individuals. In this regard, it is important to stress that in this work, we are concerned with superior rewiring rules that lead to group dominance in competitions under various conditions. In this sense, our work is different from previous attempts in which all individuals adopt the same rewiring rules^26,28,42^ and in which competitions are performed on multiple layers of networks^25,46^.

Our results show that open triads, especially typical types of open triads involving the same opinions, play a key role in group expansion and dominance. In fact, the role of triads and their variants has always been an interesting area of network science and social dynamic systems. Studies on triads are also known to strike a structural balance between complete graphs^73,74^, network patterns or motifs, and positive and negative relationships^75,76^. Interdisciplinary studies on triads focus on two main issues: static network structures^77^ (e.g., link predictions and recommendations^78,79^, individual performance^80^ and hormone-behaviour relationships^81^, and brokerage structures or trading relationships^82,83^) and dynamic network processes (e.g., community stability and growth^84^ and sexually transmitted diseases^85^). For example, ref.^84^. considers triads among community members and finds that a large density of closed triads (triangles) is negatively related to the growth of communities. Therefore, in these cases, triads can be regarded as playing a fundamental role in network structures, which affect the robustness of social dynamic systems.

Our results show that Rule II on opinion homophily rewiring is superior when applied to competitions under a balanced initial proportion and under unbalanced proportions with deterministic/indeterministic rewiring rates or initial proportions. Rule II is advantageous in that it strives to limit exposure of “your” opinion group to influence from “other” groups. This helps minorities better survive despite conforming to pressures from the majority or majorities to suppress the minority without being exposed to (counter) effects from the minority. This result relates to issues of cultural differentiation, cooperation and conflict. For example, Axelrod’s model^3^ shows that cultural convergence can be achieved among distinct cultural groups through mechanisms of homophily and social influence. Centola’s model^14^ introduces another factor, network homophily, through which network structures co-evolve with cultural interactions, and their coevolution model explains stable/unstable cultural diversity or cultural convergence in a specific region of parameter space. Our model also takes the following three mechanisms into account: homophily (Rule II), social influence (Voter model) and network topology coevolution. Our results show that homophily stabilizes a (sub-)culture against outside influences and that this causes Rule II to fare better against another group not using this rule. However, under opinion group-levelled conditions, interactions occur between individual behaviours and opinion group-levelled behaviours. On the one hand, the behaviours of individuals are guided by faith, as individuals may share similar behaviours and tend to join the same groups (e.g., a certain political party or religion), which reflects effects of homophily, social influence, etc.; on the other hand, doctrines or group rules can guide and constrain individual behaviours. Relative to current cultural differentiation models, group-levelled rewiring affects competitive group strategies more.

The present work serves as an abstract representation of empirically blurry situations involving the use of single and basic rewiring behaviours as a group strategy. (1) Here we assume that when someone asks to establish a rewired connection, the other individual does not refuse by default, i.e., a refusal ratio of 0%. In a more general case, the refusal rate can be related to other factors, such as group strategies or individual heterogeneity, and the dynamic processes involved can vary. During group competition, whether to rise the refusal ratio from the opposite opinions or accept more may affect the influence from rewiring rules. This reflects a limitation of our models. (2) A generalizing task would involve considering opinion groups that adopt mixed group behaviours as a compound group strategy or considering the different ways in which a certain preference can manifest. For example, homophily-based rewiring can involve rewiring to nodes of the same opinion with no other biases (Rule II), rewiring to nodes of the same opinion with high degrees (rewiring based on both homophily and high degrees), rewiring to nodes of the same opinion and with similar local structures (rewiring based on both opinion and network homophily), etc. Cases of network structure-based rewiring serve as another example. (3) Moreover, some individuals examined in this work were initially born with a given opinion, while others showed no initial preference. The ways in which opinion groups interact with individuals with no opinions also present several challenging issues.

## Electronic supplementary material

Supplementary Material
