# Supplementary material for: Group-based rewiring rules of binary opinion competition dynamics
Source: Sci Rep. 2018 Sep 26;8:14423. doi: 10.1038/s41598-018-32678-7 (PMC6158185; doi:10.1038/s41598-018-32678-7)
Supplement: Supplementary file 1 — Supplementary Material [file 41598_2018_32678_MOESM1_ESM.docx]

**Group-based rewiring rules in binary opinion competition dynamics**

**SUPPLEMENTARY MATERIALS**

Cheng Jin,^1,2^ Chunji Yin,^3^ Xiaogang Jin,^1, *^ Yong Min,^4^ Yixiao Li,^5^ Nuole Chen,^6^ and Jiaxuan Huang^1^

^1^*Institute of Artificial Intelligence, College of Computer Science & Technology, Zhejiang University, 310027, Hangzhou, China*

^2^ *Tencent Technology (Shenzhen) Co., Ltd., 518057, Shenzhen, China*

^3^*State Key Lab of CAD&CG, Zhejiang University, 310058, Hangzhou, China*

^4^*College of Computer Science, Zhejiang University of Technology, 310023, Hangzhou, China*

^5^*School of Information, Zhejiang University of Finance and Economics, 310018, Hangzhou, China*

^6^*Department of Political Science, University of Illinois at Urbana-Champaign, 61820, Urbana, United States*

*Corresponding author: [xiaogangj@cise.zju.edu.cn](mailto:xiaogangj@cise.zju.edu.cn)

Contents

[APPENDIX A: BASIC CASES FOR BINARY OPINION COMPETITION UNDER ONE REWIRING RULE 2](#_Toc517466505)

[APPENDIX B: PANORAMIC VIEW OF OPINION COMPETITION UNDER PAIRS OF REWIRING RULES, AND THE RESPONSE FOR THE MAJORITY WITH A GIVEN MINORITY 2](#_Toc517466506)

[APPENDIX C: RESPONSE OF DENSITY IN EDGES 0-1 TO A DENSITY OF 1S UNDER RULE I VS RULE III 3](#_Toc517466507)

[APPENDIX D: CONJECTURE FOR EVOLUTION IN CATEGORY I 3](#_Toc517466508)

[APPENDIX E: DETAILS OF THE AME METHOD 4](#_Toc517466509)

[REFERENCES 7](#_Toc517466510)

[FIGURE LEGENDS 7](#_Toc517466511)

[TABLES 11](#_Toc517466512)

**APPENDIX A: BASIC CASES FOR BINARY OPINION COMPETITION UNDER ONE REWIRING RULE**

When both opinion groups adopt the same rewiring rule, all the individuals use the same rewiring rule, and the problem for this case degenerates into previous work on rewiring rules at the individual level. These situations are the basic case for opinion competition under group strategies.

In a balanced initial opinion proportion ($a=0.5$, see Figs. S1(a)-(c)), under a low rewiring rate *w*, the trend for coexistence is near 0% and the trend for either opinion group to dominate is approximately 0.5. Under a high rewiring rate, the final opinion proportion is close to the initial shares, both groups could not merge to the other side, and they coexist. The phase transition from one opinion dominants to the coexistence of two opinions can be seen more clearly from the dotted auxiliary line of proportion of the final minority. The phase transition is continuous for Rule I vs Rule I, and is discrete for Rule II vs Rule II (with a critical point *w* near 0.4) and for Rule III vs Rule III (with a critical point *w* near 0.8). One difference between Rule III and the other two is that before the coexistence phase, the minority under Rule I or Rule II is nearly distinct (close to 0), while the minority survives in Rule III with a relative large share (over 0.2). Rule I or Rule II are conducive to form a connected component, while Rule III could leave many fragmented small components. More details of Rule I vs Rule I and Rule II vs Rule II can be seen in^1^, as they are the exact rewire-to-random model and rewire-to-same model discussed in that work.

**APPENDIX B: PANORAMIC VIEW OF OPINION COMPETITION UNDER PAIRS OF REWIRING RULES, AND THE RESPONSE FOR THE MAJORITY WITH A GIVEN MINORITY**

A full view for the win rates of opinion 1 with $w\in(0,1)$ and$a\in(0,1)$ is shown in Fig. S2.

This view also gives the proper response for the majority when given the choice of the minority. As Figs. S3(a)-(c) show, no matter what the rewiring rule minority choose when *w* is indeterministic, the best response for the majority is always Rule II. And as Figs. S3(d)-(f) show, with indeterministic $a\in(0.5,1)$, Rule II is also the best response in most situations with a given *w*. And when both *a* and *w* are indeterministic, Rule II is still the superior group strategy for the initial majority no matter what rule the minority choose.

**APPENDIX C: RESPONSE OF DENSITY IN EDGES 0-1 TO A DENSITY OF 1S UNDER RULE I VS RULE III**

The evolution of Rule I vs Rule III (see Fig. S4) shows some difference with the evolution of Rule I vs Rule II stated in Fig. 5. The former shows less sensitivity on rewiring rate *w* and the arch collapse with $w\approx0.7$. Besides, the right absorbing state $\theta_{c_{2}}$ shifts away from 1.0, and the left absorbing state $\theta_{c_{1}}$ shifts away from 0.0. However, both the two cases meet that the maximum points $Z_{w}$ on the arch show a bias on $Z_{w_{1}}<0.5$.

**APPENDIX D: CONJECTURE FOR EVOLUTION IN CATEGORY I**

Evolutions in Category I are nontrivial and they share similarities with a finite Markov chain with two absorbing states. In this appendix, we formulate the conjecture for this evolution by using simulation results and analogs to the known theorem and results.

**Theorem.** If the voter model on a torus in $d\geq3$ starts from a product measure with density $p$ (the opinions of nodes at time *t* are independent and equal to 1 with probability *p*). Then, at time $Nt$, it looks locally like $v_{\theta(t)}$ where the density $\theta\left( t \right)$ changes according to the Wright-Fisher diffusion process

$d\theta_{t}=\sqrt{\beta_{d}2\theta_{t}(1-\theta_{t})}dB_{t}$） (1)

$\beta_{d}$ is the probability that two random walks starting from neighboring sites never hit each other.

The voter model on the torus does not have a nontrivial stationary distribution, but it does have a one-parameter family of “quasi-stationary distributions” that look locally like that; and the quantity under the square root in the Wright-Fisher diffusion is, according to the results of Holley and Liggett^2^, the expected value of $N_{10}/M$ under $\nu_{\theta(t)}$.

Similarly, simulation results of evolutions under Rule I vs Rule I or Rule II vs Rule II have been formulated by Durrett^1^ as conjectures.

**Durrett’s conjecture**. In the rewire-to-random model (Rule I vs Rule I), if $w<w_{c}(0.5)$ and $\nu\left( w \right)<a\leq0.5$, then starting from a product measure with a density $a$ of 1s, the evolving voter model converges rapidly to a quasi-stationary distribution $\nu_{w,a}$. At time $tN$, the evolving voter model looks locally like $\nu_{w,\theta(t)}$ where the density changes according to a generalized Wright-Fisher diffusion process

$d\theta_{t}=\sqrt{\left( 1-w \right)[c_{w}\theta_{t}\left( 1-\theta_{t} \right)-b_{w}]}dB_{t}$ (2)

until $\theta_{t}$ reaches $\nu_{w}$ or $1-\nu_{w}$, given $\theta(t)$ as ${N_{1}}/N$ at time *t* and $\left( \nu_{w}, 1-\nu_{w} \right)$ as the support interval where the arch has positive values. When $b_{w}=0$, the behavior could describe the rewire-to-same model (Rule II vs Rule II), and $w_{c}$ is independent of the initial density$a$. For $w<w_{c}$, the final density$\rho\approx\nu\left( w \right)=0$; for $w>w_{c}$, the final density$\rho\approx a$; and for $w=w_{c}$, the final density is within the range [0, *a*].

Durrett’s conjecture could describe the cases when a single rewiring rule such as Rule I or Rule II exists for both opinion groups, while it is not applicable to evolution under non-unique rewiring rules that also follow quasi-stationary distributions. Using the simulation results of evolutions under Rule I vs Rule II or Rule I vs Rule III, we formulated a conjecture that can describe evolution under either one single rewiring rule or two non-unique rewiring rules.

**Our extended conjecture.** For an opinion competition model under Rule I vs Rule I, Rule I vs Rule II, Rule I vs Rule III, and Rule II vs Rule II, the density changes to

$d\theta_{t}=\sqrt{\left( 1-w \right)(a_{w}\theta_{t}^{3}+b_{w}\theta_{t}^{2}+c_{w}\theta_{t}+d_{w})}dB_{t}$ (3)

until $\theta_{t}$ reaches $\theta_{c_{1}}$ or $1-\theta_{c_{2}}$. This behavior fits all the evolutions in Category I. The detailed final proportions of minority are listed in Table SI (the evolutions bound on the arch, $\theta_{c_{1}}<a\leq0.5$).

**APPENDIX E: DETAILS OF THE AME METHOD**

The original AME method estimates opinion dynamics in an infinite population on uncorrelated static networks^3,4^. It is extended for approximation to dynamic networks in Ref^1^, which solves a situation called the rewire-to-random model (Rule I vs Rule I) and the rewire-to-same model (Rule II vs Rule II). The executable code for the AME is given in Ref.^5^.

However, previous work does not consider the opinion competition under non-unique rewiring rules. And, in these cases, the AME framework meets its limitations after the initial transient. In this appendix, we give the AMEs and explain the way we use states calculated in the critical stage to approximate evolutions in correlated dynamics such as Rule I vs Rule II, as well as Rule I vs Rule III.

**Rule I (opinion 0) vs Rule II (opinion 1)**

Following the ideas presented in Section IV.A.2, when considering the case of Rule I vs Rule II, at time *t*, $\bar{S}_{k,m}$ relates to $\bar{S}_{k,m}$, $\bar{S}_{k,m+1}$, $\bar{S}_{k+1,m+1}$, $\bar{S}_{k-1,m-1}$, $\bar{S}_{k-1,m}$, and $\bar{I}_{k,m}$. With a probability *w*, rewiring breaks the connection between nodes *x* and *y* and brings a new edge to connect to *x* or *y*. This happens with the exact equations$-\left( 2-a \right)m\bar{S}_{k,m}+\left( 1-a \right)\left( m+1 \right)\bar{S}_{k,m+1}+\left( m+1 \right)\bar{S}_{k+1,m+1}$. With probability *w*, nodes *x* or *y* may influence the other by a voting step. This happens with the exact equations$wN_{01}\left[ -2\bar{S}_{k,m}+\bar{S}_{k-1,m-1}+\bar{S}_{k-1,m} \right]/N$. And finally, the opinion of *y* may be changed by imitating one of its neighbors *z≠x*. The approximate equation for this is $\left( 1-w \right)\left[ -m\bar{S}_{k,m}+\left( k-m \right)\bar{I}_{k,m} \right]+\left( 1-w \right)[-\beta^{S}\left( k-m \right)\bar{S}_{k,m}+\beta^{S}\left( k-m+1 \right)\bar{S}_{k,m-1}-\gamma^{S}m\bar{S}_{k,m}+\gamma^{S}(m+1)\bar{S}_{k,m+1}]$.

By considering all the possible changes, one arrives at the following AME for events in $\bar{S}_{k,m}$:

$\frac{d}{dt}\bar{S}_{k,m}=w\left\{ -\left( 2-a \right)m\bar{S}_{k,m}+\left( 1-a \right)\left( m+1 \right)\bar{S}_{k,m+1}+\left( m+1 \right)\bar{S}_{k+1,m+1} \right\}+wN_{01}\left[ -2\bar{S}_{k,m}+\bar{S}_{k-1,m-1}+\bar{S}_{k-1,m} \right]/N+\left( 1-w \right)\left[ -m\bar{S}_{k,m}+\left( k-m \right)\bar{I}_{k,m} \right]+\left( 1-w \right)[-\beta^{S}\left( k-m \right)\bar{S}_{k,m}+\beta^{S}\left( k-m+1 \right)\bar{S}_{k,m-1}-\gamma^{S}m\bar{S}_{k,m}+\gamma^{S}(m+1)\bar{S}_{k,m+1}]$ (4)

Using the same idea, we get the equations for events in $\bar{I}_{k,m}$:

$\frac{d}{dt}\bar{I}_{k,m}=w\left\{ -2(k-m)\bar{I}_{k,m}+\left( k-m+1 \right)\bar{I}_{k-1,m-1}+\left( k-m+1 \right)I_{k,m-1} \right\}+wN_{01}\left[ -\bar{I}_{k,m}+\bar{I}_{k-1,m-1} \right]/[Na]+\left( 1-w \right)\left[ -(k-m)\bar{I}_{k,m}+m\bar{S}_{k,m} \right]+\left( 1-w \right)[-\beta^{i}\left( k-m \right)\bar{I}_{k,m}+\beta^{i}\left( k-m+1 \right)\bar{I}_{k,m-1}-\gamma^{i}m\bar{I}_{k,m}+\gamma^{i}(m+1)\bar{I}_{k,m+1}]$ (5)

where

$$\beta^{S}=\frac{\sum_{k,m} \left( k-m \right)m\bar{S}_{k,m}}{\sum_{k,m} \left( k-m \right)\bar{S}_{k,m}}=\frac{N_{001}}{N_{00}}$$

$$\gamma^{S}=\frac{\sum_{k,m} \left( k-m \right)^{2}\bar{I}_{k,m}}{\sum_{k,m} \left( k-m \right)\bar{I}_{k,m}}=\frac{N_{010}}{N_{01}}+1$$

$$\beta^{I}=\frac{\sum_{k,m} m^{2}\bar{S}_{k,m}}{\sum_{k,m} m\bar{S}_{k,m}}$$

$$\gamma^{I}=\frac{\sum_{k,m} \left( k-m \right)m\bar{I}_{k,m}}{\sum_{k,m} m\bar{I}_{k,m}}$$

Here, $\beta^{S}$ gives the expected number of edges 0-0 to edges 0-1 and $\gamma^{S}$ gives the opposite expected number of edges 0-1 to edges 0-0; similarly, $\beta^{I}$ gives the expected number of edges 1-1 to edges 0-1 and $\gamma^{I}$ the expected number of edges 0-1 to edges 1-1. We use the fact that in the initial phase modeled by these differential equations, $N_{1}\approx Na$ and $N_{0}\approx N(1-a)$. And $\sum_{k,m} m\bar{S}_{k,m}=N_{01}$, $\sum_{k,m} (k-m)m\bar{S}_{k,m}=N_{01}$. All the equations should meet $N_{11}+2N_{10}+N_{00}=M$.

The approximations for the arches reported in Fig. 6 in the main text are obtained by numerically solving the system in the steady state. The MATLAB odeset function was used, starting with a Poisson degree distribution of mean degree λ=4, nodes independently assigned the value 1 with probability *a*, and the equations cut off at maximum degree of *K*= 50 (the results are not appreciably affected by increasing *K*).

**Approximation to correlated, finite networks based on AMEs**

The evolution begins with an initial transient stage and then reaches a special stage where a quasi-stationary distribution exists. The approximation from AMEs for basic cases Rule I vs Rule I and Rule II vs Rule II can describe the whole process well^1^, but here we show that in Rule I vs Rule II, the original results from the AMEs show limitations on describing the entire evolution process (see Fig. S5(a), (c)). This is due to the limitation of using an infinite population to simulate a finite population on dynamics correlated to network structures. However, the numerical results show that, under the same rewiring rate, the initial transient stage (a stage where the density of discordant edges drops while the density of either opinion remains stable) for various initial densities *a* is nearly the same (see Fig. S5(b)) on time steps. In addition, the numerical results of the initial stage match the simulation results (see Fig. S5(c)) well. Thus, although AMEs do not meet the entire evolution under Rule I vs Rule II, they are indeed consistent with simulation results for the initial transient parts.

Therefore, our approximate calculus based on AMEs contains three steps: (1) record the original results calculated by the AMEs; (2) find the critical stage from the initial transient to the following dynamic process; and (3) fit the states in critical stages as the estimation from AMEs (see Fig. S5(d)). One should notice that when *a* is large, the AME approximation is less effective (see Fig. S5(d)). So, we use more cases in *a*<0.5 to fit the evolution of Rule I vs Rule II. This extended way can describe all the evolutions in Category I.

**Rule I vs Rule III**

For Rule I vs Rule III, the changes for $\bar{S}_{k,m}$ are exactly the same as in the case of Rule I vs Rule II. The changes for $\bar{I}_{k,m}$ are as follows:

$\frac{d}{dt}\bar{I}_{k,m}=w\left\{ -\left( 1+v \right)(k-m)\bar{I}_{k,m}+v\left( k-m+1 \right)\bar{I}_{k,m-1}+\left( k-m+1 \right)I_{k+1,m} \right\}+wN_{01}\left[ -2k\bar{I}_{k,m}+\left( k-1 \right)\bar{I}_{k-1,m}+\left( k-1 \right)I_{k-1,m-1} \right]/D+\left( 1-w \right)\left[ -(k-m)\bar{I}_{k,m}+m\bar{S}_{k,m} \right]+\left( 1-w \right)[-\beta^{i}\left( k-m \right)\bar{I}_{k,m}+\beta^{i}\left( k-m+1 \right)\bar{I}_{k,m-1}-\gamma^{i}m\bar{I}_{k,m}+\gamma^{i}(m+1)\bar{I}_{k,m-1}]$ (6)

where $v$ is the proportion of degrees for nodes holding opinion 1: $v=\sum_{k,m} kI_{k,m}/(\sum_{k,m} kI_{k,m}+\sum_{k,m} kS_{k,m})$ and $D$ is the total number of degrees $D=\sum_{k,m} kI_{k,m}+\sum_{k,m} kS_{k,m}$. Again, we generate our predictions by numerically solving the differential equations and find the critical stage. Here we set *K*=100 and λ = 4, to take into account the high degrees and the accuracy of the calculus. The predictions for the arch are given in Fig. S6.

**REFERENCES**

1. Durrett, R. *et al.* Graph fission in an evolving voter model. *Proc. Natl. Acad. Sci.* **109,** 3682–3687 (2012).

2. Holley, R. A. & Liggett, T. M. Ergodic Theorems for Weakly Interacting Infinite Systems and the Voter Model. *Ann. Probab.* **3,** 643–663 (1975).

3. Gleeson, J. P. High-Accuracy Approximation of Binary-State Dynamics on Networks. *Phys. Rev. Lett.* **107,** 068701 (2011).

4. Gleeson, J. P. Binary-State Dynamics on Complex Networks: Pair Approximation and Beyond. *Phys. Rev. X* **3,** 021004 (2013).

5. Gleeson, J. P. Octave/MATLAB code for solving the differential equations arising from the approximate master equations, pair approximations, and mean-field theories. *Octave/MATLAB code for solving the differential equations arising from the approximate master equations, pair approximations, and mean-field theories* (2017). Available at: http://www3.ul.ie/gleesonj/solve_AME/index.htm. (Accessed: 17th September 2017)

**FIGURE LEGENDS**


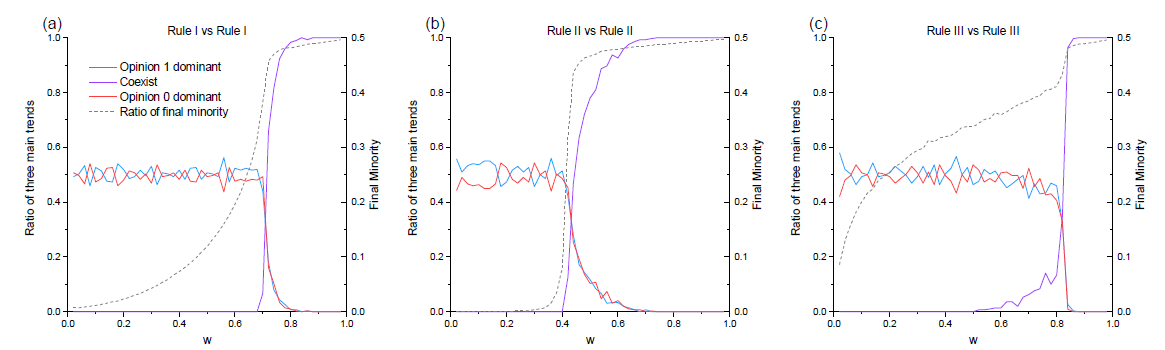


**Figure S1** Binary opinion competition under a single rewiring rule. Corresponding to a rewiring rate *w*, the blue line denotes that opinion 1 dominates, the red line denotes opinion 0 dominates, and the purple line denotes coexistence. The dotted line is the ratio of the final minority. (a) The three main trends under case Rule I vs Rule I. (b) Rule II vs Rule II. (c) Rule III vs Rule III. Here, *N*=2000, *M*=4000, and for each *w* in each case, the number of simulations is 300.


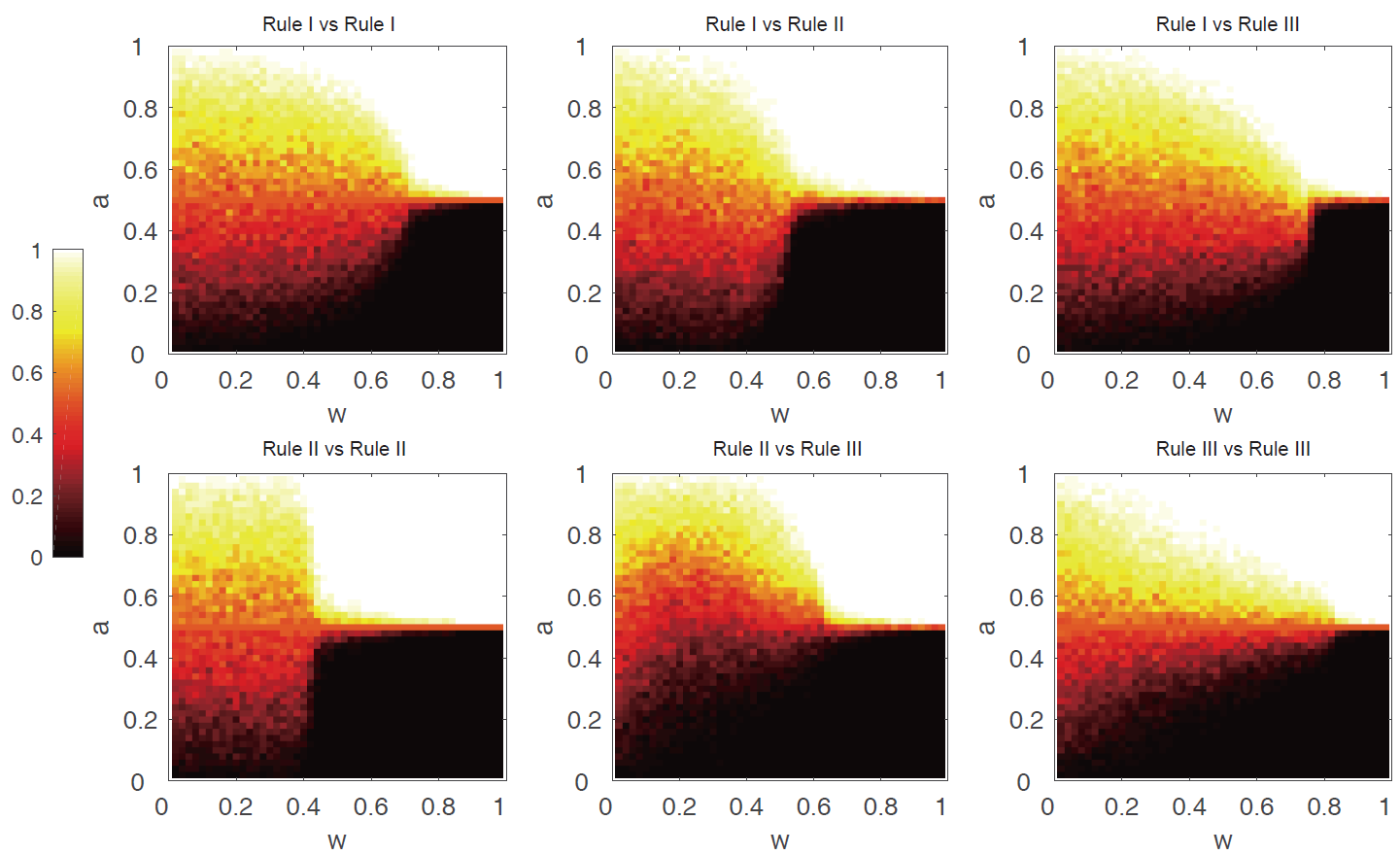


**Figure S2** Responses of the group holding opinion 1 under various conditions. The color is for the win rate of 1s adopting Rule X (X can be I, II, III) with a rewiring rate *w* and initial density *a*. Here *N*=2000, *M* = 4000, and for each *w* and *a*, there are 100 simulations. The win rate is the probability that the final density of opinion 1s is over 0.5.


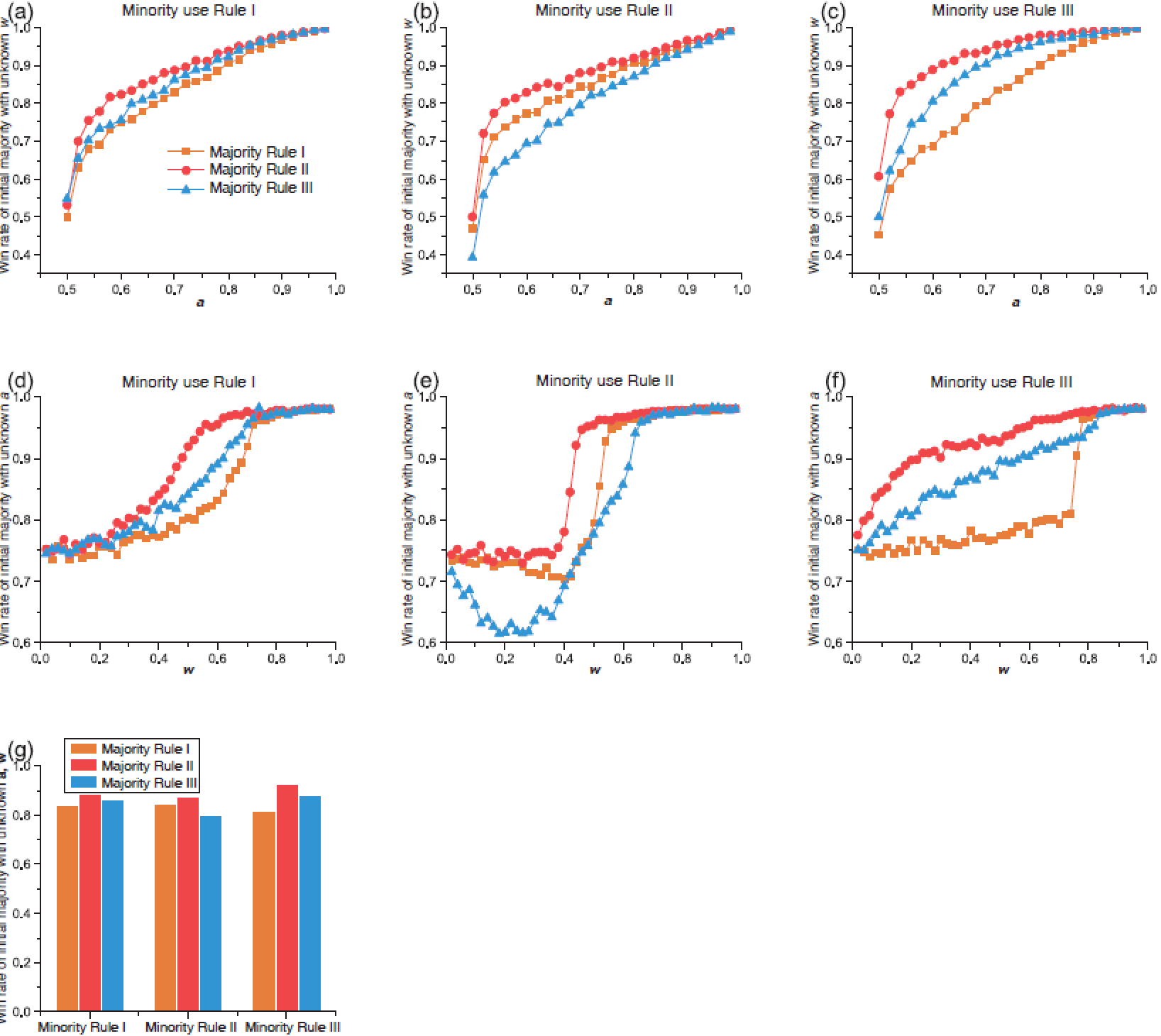


**Figure S3** Responses for the majority. (a) Win rate for the majority with indeterministic *a* or *w*. This is the average win rate for $w\in(0,1)$ and $a\in(0,0.5)$. (b)-(d) Win rate of the majority with a given majority and *a*, but an indeterministic *w*. Orange means that the majority adopt Rule I, red is for adopting Rule II, and blue is for adopting Rule III. (b) Minority adopt Rule I. (c) Minority adopt Rule II. (d) Minority adopt Rule III. (e)-(g) Win rate of the majority with a given minority and *w*, but an indeterministic *a*. (e) Minority adopt Rule I. (f) Minority adopt Rule II. (g) Minority adopt Rule III.


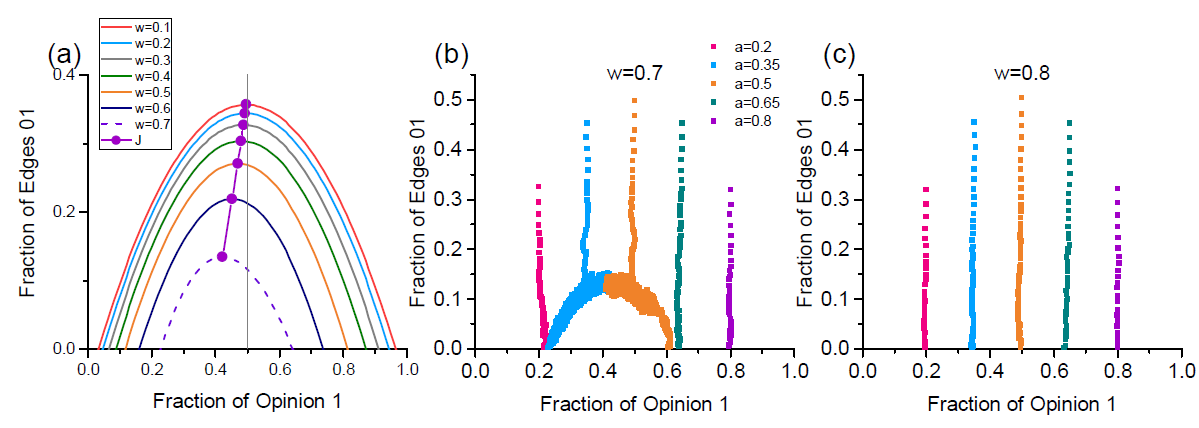


**Figure S4** Evolution for competing cases under Rule I vs Rule III. (a) Observed arches for Rule I vs Rule III with *w* = 0.1, 0.2, 0.3, 0.4, 0.5, 0.6, and 0.7. The purple line is the trend of the maximum points on these arches. (b) Observed evolution when *w*=0.5. (c) Observed evolution when *w*=0.6. The specified parabolas are fits to simulation data with *N*=2000, *M*=4000.


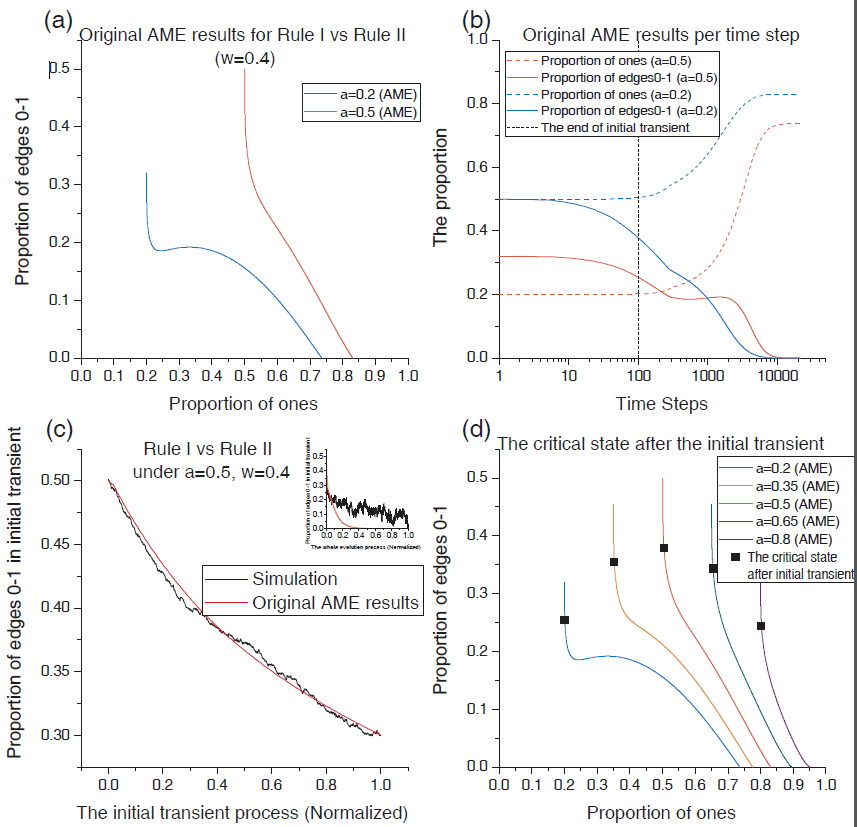


**Figure S5** Results of the approximate master equation (AME) under asymmetric rewiring Rule I vs Rule II. (a) Results directly solved using the AME with *a*=0.2 or 0.5 and *w*=0.4. (b) The proportion of opinion 1s and edges 0-1 per time step. The dotted auxiliary line is the stage between stable proportions of 1s (initial transition) to changed proportion of 1s (the later process) with *a*=0.2 or 0.5 and *w*=0.4. (c) The normalized initial transient process for simulation results and results directly solved by AME. And the upper coordinate is the normalized results for the whole evolution process with *a* = 0.5 and *w*=0.4. (d) Black squares are the critical states with *a*=0.2, 0.35, 0.5, 0.65, 0.8, and color lines are the results directly solved using the AME.


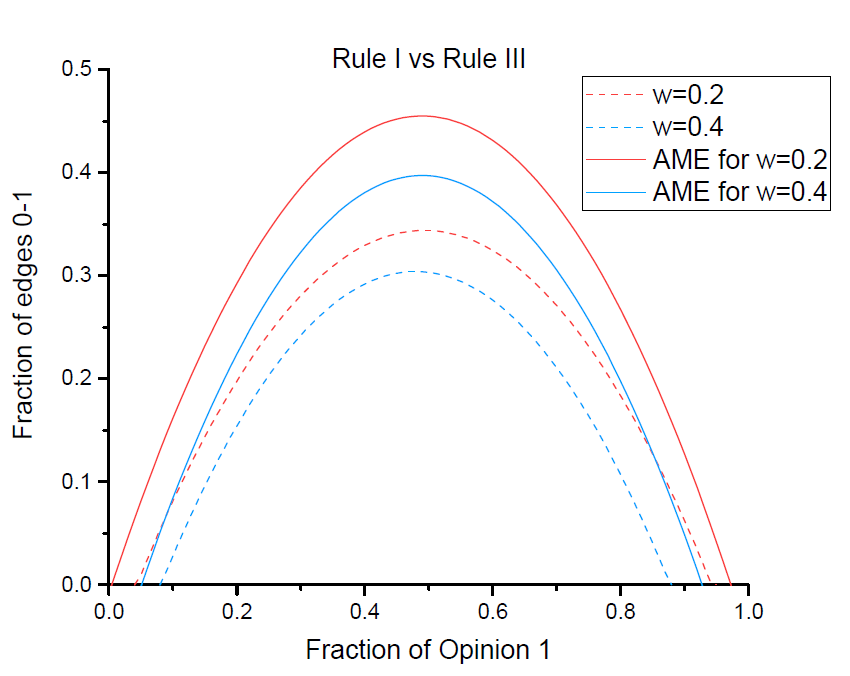


**Figure S6** Arches computed by the approximate master equation (AME) versus simulation under Rule I vs Rule III with $w=0.2\left( \mathrm{red} \right), 0.4\left( \mathrm{blue} \right)$. The solid line shows the results solved and fitted using the AME, while the dotted line shows the simulation results.

**TABLES**

**Table SI** Parameters for evolution under different pairs of rewiring rules

| Parameters | Corresponding pairs  of rewiring rules | Final opinion density $\rho$  (suppose $\nu\left( w \right)<a\leq0.5$ and $\theta_{c_{1}}\left( w \right)<a\leq0.5$) |
| --- | --- | --- |
| $a_{w}=0$  $b_{w}+c_{w}=0$  $d_{w}=0$ | Rule II vs Rule II | If $w<w_{c}$, $\rho\approx v\left( w \right)=0$  If $w>w_{c}$, $\rho\approx a$  If $w=w_{c}$, $\rho\in[0,a]$ |
| $a_{w}=0$  $b_{w}+c_{w}=0$  $d_{w}\neq0$ | Rule I vs Rule I | If $w<w_{c}\left( 0.5 \right)$, $\rho(w,0.5)\approx\nu(w)$  If $w>w_{c}(0.5)$, $\rho(w,0.5)\approx a$  If $w=w_{c}(0.5)$, $\rho\left( w,0.5 \right)\in[\nu(w),a]$ |
| $a_{w}\neq0$  $d_{w}\approx0$ | Rule I vs Rule II  or Rule I vs Rule III | If $w<w_{c}(a)$, $\rho\approx\theta_{c_{1}}(w)$  If $w>w_{c}(a)$, $\rho\approx a$  If $w=w_{c}(a)$, $\rho\in[\theta_{c_{1}}\left( w \right), a]$ |
